# Periprosthetic Fractures of the Femur during the Operation of Primary Total Hip Arthroplasties

**DOI:** 10.1055/s-0044-1779320

**Published:** 2024-01-31

**Authors:** Conrado Auer Trentini, Marcela de Andrade Balsano, Mark Deeke, Francisco José Zaniolo

**Affiliations:** 1 Serviço de Ortopedia e Traumatologia, Hospital Universitário Regional dos Campos Gerais, Ponta Grossa, PR, Brasil; 2 Serviço de Ortopedia e Traumatologia, Hospital Universitário Cajuru, Curitiba, PR, Brasil

**Keywords:** arthroplasty, hip fractures, intraoperative complications, periprosthetic fractures

## Abstract

**Objective** To determine the incidence of periprosthetic femoral fractures during the operation in primary hip arthroplasties and correlate them with the inherent risk factors associated with patients, implants, and the diagnosis of coxarthrosis and/or femoral neck fractures.

**Methods** Cross-sectional study, with retrospective analysis of medical records and image exams of patients operated between 2014 and 2019. The variables analyzed followed those proposed by the world literature, namely: age, sex, Dorr index, surgical indication, Vancouver classification, location, type of fixation (cemented or non-cemented), implant model used, intraoperative diagnosis, and corresponding treatment approach. All surgeries used the same posterolateral access route and were performed by the same group of surgeons.

**Results** Within the sample of 2,217 arthroplasties (2,154 patients), 12 fractures (0.56%) were identified in 12 patients. The sample consisted of 8 females and 4 males, with an average age of 62.53 years. In all diagnosed cases, protective cerclages were added at the level of the lesser trochanter and/or the greater trochanter, and, in 3 cases, there was a change from uncemented to cemented femoral stems and only 1 required revision.

**Conclusion** Data Analysis Allows Us To State That The Risk Of Intraoperative Periprosthetic Fracture Is Greater In Women And With The Use Of Cementless Stems. The Occurrence Of These In This Study Had A Rate Of Less Than 1%.

## Introduction

Total hip arthroplasty (THA) is a common and successful surgical procedure in the treatment of patients with osteoarthritis or hip fractures, whose main objective is to provide pain relief and restore joint biomechanics, substantially improving the quality of life of the patient. 1

Among the complications resulting from THA, periprosthetic fracture is mentioned. It is considered a rare complication that, when not diagnosed during surgery, requires complex, clinically relevant treatment and is generally associated with an unfavorable outcome, such as the need for early revision arthroplasty and poor functional recovery. 2 For these reasons, it is important to identify which predisposing factors are present in the cases to be operated on so that we can reduce the risks of this complication. 3

Intraoperative fractures in primary arthroplasties can be observed during surgery and are related to the type of implant, uncemented prostheses, incompatibility between the rasp and the implant, joint interface, gender and/or defects created during the osteotomy or milling of the femoral canal. 4 The fracture diagnosis can be made through direct visualization of the line, a change in sound during the impaction of reamers, a sudden alteration in resistance during the implantation of the definitive femoral component, or through the immediate postoperative x-ray. 5

After diagnosis, it is important to classify the fractures as this will guide the treatment strategy. To this end, we used the Vancouver classification for intraoperative femoral fractures, which takes into account the stability of the implant. Type A occurs in the proximal metaphysis, type B occurs in the diaphysis, and type C occurs distal to the shaft. They can further be subdivided into type I (perforation), type II (non-displaced fracture line), and type III (unstable or displaced). 4

Dorr et al. 6 classified the conformation of the proximal femur into types A, B, and C based on the thickness of the cortices and the shape of the spinal canal. Patients with type C femoral pain had a 6.5 times higher risk of postoperative periprosthetic fracture compared to those with type A, while female patients had a 1.3 times higher risk of experiencing this complication. 7 Considering intraoperative fractures, 86% occur during the use of cementless stems in patients with good bone quality, Dorr A or B. 8

The consequences of intraoperative periprosthetic fractures are varied resulting in higher readmission rates, financial burden, inferior functional outcome, increased revision rates, and increased mortality. Given this scenario, the importance of diagnosing these fractures intraoperatively is highlighted, since the treatment is highly effective and low complexity. 9

In this scenario, the present study aims to determine the incidence of periprosthetic femur fractures during the operative period in primary hip arthroplasties and correlate it with the risk factors inherent to patients, implants, and the diagnosis of coxoarthrosis and/or fracture of the femoral neck.

## Materials and Methods

This is a retrospective cross-sectional study, based on medical records and imaging exams from the archives of 2 tertiary hospitals from 01/01/2014 to 12/31/2019. All patients undergoing primary total hip arthroplasty via posterolateral access during the study period and with a period of at least 1 year of postoperative follow-up were included. This research was submitted for analysis by the Research Ethics Committee of Hospital Universitário Cajuru- Curitiba/PR and Hospital Marcelino Champagnat- Curitiba/PR.

Patients undergoing revision arthroplasty, those with previous surgeries on the affected hip, resection arthroplasty, patients who missed follow-ups, and cases related to pathological fracture were excluded.

The variables analyzed followed those suggested in the literature and were age, sex, Dorr index, surgical indication, Vancouver fracture classification, location, type of femoral fixation (cemented or uncemented), implant model used, intra or postoperative diagnosis and corresponding treatment approach ( Table 1 ). All cases were performed by a surgical team comprising 5 members, 4 of whom were experienced senior surgeons with over 6 years of expertise, and 1 junior surgeon with 2 years of experience at the beginning of the study.

**Table 1:** Cases

Age	Sex	Door	Indication	Vancouver	Location	Cemented stem	Model used	Diagnosis	Treatment
74	F	C	Fracture	A2	GT	No	Stryker- Accolade II (Portage, MI, USA)	Yes	Conversion + cerclage
56	M	B	Osteoarthritis	A2	GT	No	Zimmer-ML (Warsaw, IN, USA)	Yes	Cerclage
73	F	A	Osteoarthritis	A2	Calcar	No	Stryker- Accolade II (Portage, MI, USA)	Yes	Cerclage
74	F	B	Fracture	A2	Calcar	No	Jhonson- Corall (São Paulo, SP, BR)	Yes	Cerclage
65	F	B	Osteoarthritis	A2	Calcar	No	Jhonson- Corall (São Paulo, SP, BR)	Yes	Conversion + cerclage
78	F	A	Osteoarthritis	A2	GT	No	Aesculap- Bicontact (Tullingen, GER)	No	RATQ
67	F	B	Osteoarthritis	A2	Calcar	No	Jhonson- Corall (São Paulo, SP, BR)	Yes	Conversion + cerclage
47	M	A	Osteoarthritis	A2	Calcar	No	Aesculap- Bicontact (Tullingen, GER)	Yes	Cerclage
83	M	A	Osteoarthritis	A2	Calcar	No	Jhonson- Corall (São Paulo, SP, BR)	Yes	Conversion + cerclage
62	M	B	Osteoarthritis	B2	Diaphysis	Yes	Baumer-Alpha (Mogi Mirim, SP, BR)	Yes	Cerclage
32	F	A	Osteoarthritis	A2	Calcar	No	Aesculap- Bicontact (Tullingen, GER)	Yes	Cerclage
40	F	B	Osteoarthritis	A2	Calcar	No	Aesculap- Bicontact (Tullingen, GER)	Yes	Cerclage

Abbreviations: GT, great trochanter; Conversion, replacement with cemented femoral stem, RATQ, arthroplasty revision.

The sample consisted of 2,217 arthroplasties performed on 2,154 patients, with 12 fractures (0.56%) identified. All radiographs from the immediate postoperative period, as well as subsequent follow-up imaging examinations (15th day, 4 and 6 weeks, 3 months, 6 months, and yearly), were analyzed.

The data obtained were analyzed quantitatively using the Microsoft Excel Office 2010 software (Microsoft Corporation, Redmond WA, USA) for absolute and relative frequency measurements. Comparisons between variables were carried out using the Student t test, after checking the normality and variance of the data. Statistical significance was *p* < 0.05.

## Results

12 femoral fractures that occurred intraoperatively (0.56%) were detected in a total of 2,117 hip arthroplasties in 2,054 patients over 6 years ( Table 2 ).

**Table 2:** Arthroplasties performed over a 6-year period by the group of surgeons, total number and percentage of intraoperative periprosthetic fractures

Year	Total number of surgeries	Total number of periprosthetic fractures	Percentage of periprosthetic fractures
2014	272	0	0%
2015	280	1	0.35%
2016	335	1	0.29%
2017	382	0	0%
2018	440	6	1.36%
2019	408	4	0.98%

The femoral component was cemented in 1,208 cases (57.06%) and uncemented in 909 cases (42.93%), with an increase in the frequency of use of uncemented implants over the years ( Table 3 ).

**Table 3:** Total quantity of cemented and uncemented femoral components

Year	Cemented	Uncemented
2014	156	116
2015	161	119
2016	176	159
2017	117	265
2018	179	261
2019	120	288

Eleven fractures were detected intraoperatively. In a case in which there was no diagnosis during surgery, nor was it possible to show the fracture on the immediate control x-ray, the patient developed disproportionate postoperative discomfort and pain, and, on the 13th day, he presented to the emergency room reporting severe pain associated with functional impotence without history of trauma, with the fracture being identified, which occurred intraoperatively.

Regarding cementation of the femoral component, there was 1 case (0.11%) involving the use of a cemented implant (n = 909, *p* = 0.004), and 11 cases (0.91%) involving the use of uncemented implants (n =1,208, *p* = 0.005). Of the 12 reported cases, 8 occurred in women (66.6%), and the age of the patients ranged between 32 and 83 years, with an average of 62.5 years.

**Fig. 1:**
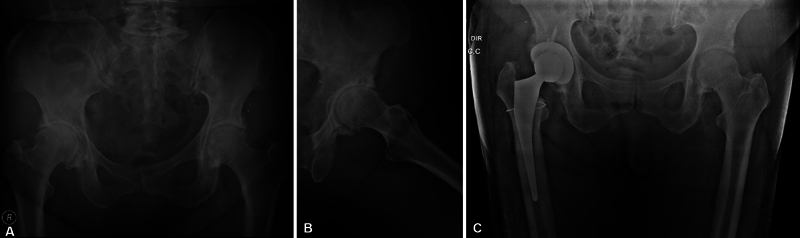
( **A-B** ) Preoperative radiograph, **C** ) Immediate control radiograph, maintenance of the nail and cerclage above the lesser trochanter. Source: researcher's collection.

Out of the 10 elective arthroplasties performed, 8 were due to arthrosis, while only 2 were prompted by femoral neck fractures. Despite being carried out by different surgeons, all procedures employed the same posterolateral access approach. The surgical technique for all femoral stems involved a sequential milling of the canal until achieving implant stability, and there were no fractures during test reductions. In 4 instances, the decision was made to replace the femoral stem with a cemented one and secure the fracture with cerclage, utilizing a 1.5-mm steel wire positioned above the lesser trochanter or, if necessary, involving the greater trochanter. In the remaining cases, the stem was retained, and treatment was administered using cerclage with a 1.5-mm steel wire, as shown in Fig. 1 .

**Fig. 2:**
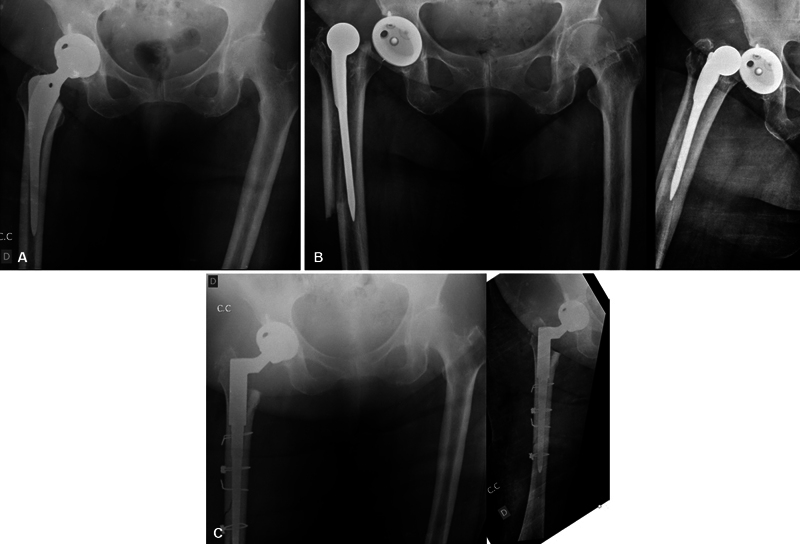
( **A** ) Immediate postoperative X-ray, ( **B** ) X-ray on the 13th day of evolution, ( **C** ) Control X-ray after review. Source: researcher's collection.

In the case in which the fracture was not identified intraoperatively but upon the patient's postoperative return, the radiography detected a fracture starting in the greater trochanter and extending to the metaphyseal region (Vancouver B2) in addition to signs compatible with implant loosening. In this case, a revision arthroplasty was performed with a distal fixation femoral stem ( Fig. 2 ).

Visual and tactile stability was achieved in all cases, and weight bearing was permitted by the patient as tolerated.

## Discussion

Intraoperative periprosthetic fracture of the femur is a complication frequently described in the literature and lacks studies with a broad sample. When not detected immediately, it leads to early implant failure, increased hospital stay, increased morbidity, and costs inherent to the procedure. 10 This has a variable incidence in the literature, being approximately 0.62% for cemented implants and 0.87% for non-cemented implants in a sample of 793,823 THAs. 11 There is currently an increase in the incidence of this event, which is related to the aging of the population and the increased use of uncemented stems. 12 13

Over the course of 6 years, we obtained an incidence of 0.11% using cemented stems and 0.91% using uncemented stems, which is compatible with the global literature. The risk approximately doubles in women. 14 Our data point to an increase in the prevalence of fractures with increasing age in women, which does not occur in men. This difference may exist because women are more affected by postmenopausal osteoporosis, which leads to a decrease in bone resistance. 15

Increasing age has already been associated with an increase in intraoperative fractures, 14 but young patients may have a greater risk of suffering calcar fractures due to the proximal femoral canal being narrower and requiring longer reaming with greater force transmitted between the bone and the instrument.

The analysis in Table 2 shows the progressive increase in the use of uncemented femoral stems instead of cemented ones, a change that has occurred over the years due to a global trend in changing the technique. Uncemented stems, when properly indicated and executed, can reduce surgical time and also the chances of pulmonary embolism resulting from the cementation process. 16 Another possible explanation for this migration is lower revision rates due to aseptic loosening. 17

The cementless stem model is the most common in THAs performed in the United States, although studies have demonstrated excellent results over the years using different femoral fixation options, a scenario that points to an increased risk of intraoperative fracture when using uncemented stems, 14 as evidenced in our data. Correct planning of the prosthesis with radiographs and transparencies is essential to reduce this risk, since there is the possibility that specific radiographic parameters indicate a greater risk of periprosthetic fracture, for example, those with Dorr type A and type B conformation. In our sample, 42% of fractures occurred in type B canals and 50% in type A canals.

Fractures of the calcar or diaphysis tend to occur during canal preparation or implant insertion, since most uncemented stems use the pressfit concept, which increases tension in the bone cortex. Among the causes of fractures related to surgical conditions inherent to the surgery that occur in the proximal region, we can mention the mismatch between the drill used in the preparation and the definitive implant, or the excessive use of force during surgical exposure and/or preparation of the femoral canal. Finally, distal fractures generally occur due to collision of the straight tip of the implant with the curved cortex of the femur. The type of fracture in which the lesser trochanter is affected occurs secondary to the propagation of microfractures that may have occurred during the primary injury, in the case of a fracture, or during osteotomy. The pattern around the stem occurs due to the incompatibility of the metaphyseal-diaphyseal junction of the stem and the canal during implantation in osteoporotic femurs. 18

The durability of the cementless stem has been shown to be greater in young patients, perhaps due to better bone quality, which reduces the risk of fractures. 19 However, the increased risk of intraoperative fracture and, consequently, its complications must be considered in relation to the longer survival of the implant, especially in patients in whom the proximal femur appears weak or in cases that require prolonged preparation and greater use of force, as in the cases Dorr A. 11

The evaluation of the nail design is something that must always be taken into consideration, as the use of wedge nails reveals a 10 times greater risk of causing intraoperative fracture when compared to first generation nails. 20 In two cases presented in the present study, simple wedge femoral implants with the *Fit-and-Fill* concept (Accolade II – Stryker Corporation, Kalamazoo, MI, USA) were used; this design is associated with a 3-fold increase in intraoperative fracture rates ( *p* < 0.001) compared to with anatomical stems, fully coated and conical or rounded. 21 Four cases presented occurred using preservation rods such as Corall (São Paulo, SP, BR) or Bicontact (Tullingmen, GER). In a study that corelates the rod model with a greater risk of fracture, the rasp model in which metaphyseal bone impaction occurs, resulting in bone preservation, would increase the tension around the canal at the time of introduction of the definitive implant, thus increasing the chances of intraoperative fractures. 22

It is important to analyze whether the indication for arthroplasty, due to fracture or osteoarthritis, interferes with the occurrence of intraoperative fractures. In our study, only 2 cases (16.6%) were indicated for neck fracture and 10 cases (83.3%) for osteoarthritis. The literature is controversial on this point. Those who advocate for the use of cemented stems use as an argument the increased risk of intraoperative fracture due to preexisting bone fragility that culminated in neck fracture; in parallel, the use of cemented stems could increase mortality due to complications resulting from femoral cementation and increased surgical time. 16

Klein et al., 23 in 2006, in a study that brought together 85 cases of neck fractures, with a mean age of 78 years, treated with cementless stem, demonstrated that there was no significant increase in the case of intraoperative fractures and that the patients showed good functional recovery. Richardson et al., 24 in 2020, with the analysis of 5,883 cases of total arthroplasty in neck fractures, compared the mortality rates in both techniques and obtained as a result a reduction in mortality rates when using cemented stems 30, 90, and 365 days after surgery. This difference is mainly due to the occurrence of intraoperative fractures and, consequently, reoperations when using non-cemented stems. Ekman et al., 25 in 2019, studied the effects of cementation in 10,677 total and partial arthroplasties, concluding that there was no difference in mortality. The literature is still very controversial on this point and requires further studies.

Considering that the biggest complication of intraoperative fracture is precisely not diagnosing it during surgery, it is important to pay attention to the signs of this complication. It is defined as a fracture identified during the surgical procedure or diagnosed on an immediate control radiograph; some signs, such as a sudden change in resistance during insertion of the definitive component, stopping of the definitive implant below the level of the osteotomy, audible noise, definitive implant with different size than planned and/or loss of stability; suggest a possible fracture. 5

In general, the treatment of acute periprosthetic fractures is related to a high rate of complications (63%) and reoperations (23%), and intraoperative diagnosis results in favorable outcome and low complexity of the procedure. The tactic consists of applying a circumferential cerclage with steel wire above the lesser trochanter and using the same implant after the cerclage with stabilization of the fracture. 9

In this study, the failure of the intraoperative diagnosis generated the need for a revision arthroplasty on the 13th postoperative day. The review of the exams showed that it was a hidden fracture, which, after intervention, evolved with consolidation of the fracture and full functional recovery. In three cases, the uncemented implant was exchanged for a cemented stem and cerclage was associated at the trochanter; this option being due to the greater femoral bone fragility, detected by the surgeon. All cases evolved with bone consolidation, full functional recovery of the patient, and without the implant loosening over the years.

When diagnosed intraoperatively and treated with the cerclage technique above the lesser trochanter, periprosthetic fracture has a high-resolution rate, and a good outcome is guaranteed. Late diagnosis leads to more complex treatment and greater chances of complications, and in these cases we should use the Vancouver classification for postoperative periprosthetic fractures, which will guide the most appropriate treatment strategy. 26

Thus, the development of skills by the surgeon, such as recognizing a specific femoral morphology, can change surgical planning and even the selection of the femoral stem. 27

This study provides information on the incidence, risks, and factors associated with intraoperative periprosthetic femur fracture during total hip arthroplasty. Understanding how it occurs and identifying possible factors that indicate such a complication is essential so that we can reduce complications, as once diagnosed intraoperatively, it has a less complex treatment and better results.

## Conclusions

Data analysis allows us to state that the risk of intraoperative periprosthetic fracture is greater in women and with the use of cementless stems. The occurrence of these in the present study had a rate of less than 1%.
