# Supplementary material for: Periprosthetic Fractures of the Femur during the Operation of Primary Total Hip Arthroplasties
Source: Rev Bras Ortop (Sao Paulo). 2024 Jan 31;59(5):e745–51. doi: 10.1055/s-0044-1779320 (PMC11624928; doi:10.1055/s-0044-1779320)
Supplement: Related texts

# Research article: Fraturas periprotéticas do fêmur no peroperatório das artroplastias totais primárias do quadril

**Keywords:** artroplastia, complicações intraoperatórias, fraturas do quadril, fraturas periprotéticas

## Abstract

**Objetivo** Determinar a incidência de fraturas periprotéticas do fêmur no peroperatório em artroplastias primárias do quadril e correlacioná-las com os fatores de risco inerentes aos pacientes, implantes e ao diagnóstico de coxoartrose e/ou fratura do colo do fêmur.

**Métodos** Estudo transversal, com análise retrospectiva de prontuários e exames de imagem de arquivos dos pacientes operados entre 2014 e 2019. As variáveis analisadas seguiram o proposto pela literatura mundial, sendo elas: idade, sexo, índice de Dorr, indicação cirúrgica, classificação de Vancouver, local, tipo de fixação (cimentada ou não cimentada), modelo do implante utilizado, diagnóstico intraoperatório e tratamento instituído. Todas as cirurgias utilizaram a mesma via de acesso póstero-lateral e foram realizadas pelo mesmo grupo de cirurgiões.

**Resultados** Dentro da amostra de 2.217 artroplastias (2.154 pacientes) foram identificadas 12 fraturas (0,56%) em 12 pacientes. A amostra foi composta por 8 pacientes do gênero feminino e 4 do gênero masculino, com média de idade de 62,53 anos. Em todos os casos diagnosticados foram adicionadas cerclagens de proteção ao nível do pequeno trocanter e ou do grande trocanter e em 3 casos houve mudança de hastes femorais não cimentadas para cimentadas e apenas 1 evoluiu com necessidade de revisão.

**Conclusão** A análise dos dados permite afirmar que o risco de fratura periprotética intraoperatória é maior em mulheres e com o uso de hastes não cimentadas. A ocorrência destas neste estudo teve taxa inferior a 1%.

## Introdução

A artroplastia total do quadril (ATQ) consiste em um procedimento cirúrgico comum e bem-sucedido no tratamento de pacientes com artrose ou fraturas do quadril, cujo principal objetivo consiste em proporcionar o alivio da dor e a restauração da biomecânica articular melhorando substancialmente a qualidade de vida do paciente. 1

Entre as complicações decorrentes de uma ATQ, cita-se a fratura periprotética. Esta é considerada uma complicação rara que quando não diagnosticada no ato operatório exige tratamento complexo, clinicamente relevante, e que geralmente está associada a um desfecho não favorável, como a necessidade de artroplastia de revisão precoce e recuperação funcional deficitária. 2 Por tais motivos, é importante identificar quais fatores predisponentes estão presentes nos casos a serem operados para que possamos diminuir os riscos desta complicação. 3

As fraturas intraoperatórias em artroplastias primárias podem ser observadas no ato cirúrgico e estão relacionadas ao tipo de implante, próteses não cimentadas, incompatibilidade entre a raspa e o implante, interface articular, gênero e ou defeitos criados durante a osteotomia ou a fresagem do canal femoral. 4 O diagnóstico da fratura pode ser dado através da visualização direta do traço, mudança no som durante a impacção das raspas, mudança súbita na resistência durante a implantação do componente femoral definitivo ou ainda através da radiografia de controle no pós-operatório imediato. 5

Após o diagnóstico, é importante classificar as fraturas pois isto guiará a estratégia de tratamento. Para tal, utilizamos a classificação de Vancouver para fraturas intraoperatórias do fêmur, que leva em consideração a estabilidade do implante. Sendo que as do tipo A ocorrem na metáfise proximal, as do tipo B na diáfise e as do tipo C ocorrem distais à haste. Elas ainda podem ser subdivididas em tipo I (perfuração), tipo II (traço de fratura sem desvio) e tipo III (instáveis ou desviadas). 4

Dorr et al. 6 classificaram a conformação do fêmur proximal em tipos A, B e C com base na espessura das corticais e no formato do canal medular. Pacientes com Dorr femoral tipo C tiveram um risco 6,5 vezes maior de fratura periprotética pós-operatória em comparação com o tipo A, enquanto os pacientes do sexo feminino tiveram um risco 1,3 vezes maior de apresentar esta complicação. 7 Considerando as fraturas intraoperatórias, 86% ocorrem durante o uso de hastes não cimentadas em pacientes com boa qualidade óssea, Dorr A ou B. 8

As consequências das fraturas periprotéticas intraoperatórias são variadas, resultando em taxas de readmissão maiores, ônus financeiro, desfecho funcional inferior, aumento das taxas de revisão e aumento da mortalidade. Diante deste cenário, ressalta-se a importância de se diagnosticar estas fraturas intraoperaoriamente uma vez que o tratamento é altamente resolutivo e de baixa complexidade. 9

Nesse cenário, este trabalho objetiva determinar a incidência de fraturas periprotéticas do fêmur no peroperatório em artroplastias primárias do quadril e correlacioná-las com os fatores de risco inerentes aos pacientes, implantes e ao diagnóstico de coxoartrose e/ou fratura do colo do fêmur.

## Materiais e métodos

Trata-se de um estudo transversal retrospectivo, baseado em prontuários e exames de imagem dos arquivos de 2 hospitais terciários no período de 01/01/2014 à 31/12/2019. Foram incluídos todos os pacientes submetidos à artroplastia total primária do quadril por via de acesso póstero-lateral no período do estudo e com um período de pelo menos um ano de acompanhamento pós-operatório. Esta pesquisa foi submetida à análise do Comitê de Ética em Pesquisa do Hospital Universitário Cajuru- Curitiba/PR e Hospital Marcelino Champagnat- Curitiba/PR.

Foram excluídos os casos de pacientes submetidos à artroplastia de revisão, pacientes com cirurgias prévias no quadril afetado, artroplastia de ressecção, pacientes que perderam consulta de acompanhamento e os casos relacionados à fratura patológica.

As variáveis analisadas seguiram as sugeridas pela literatura e foram idade, sexo, índice de Dorr, indicação cirúrgica, classificação da fratura Vancouver, local, tipo de fixação femoral (cimentada ou não cimentada), modelo do implante utilizado, diagnóstico intra ou pós -operatório e tratamento instituído ( Tabela 1 ). Todos os casos foram operados por um grupo de 5 cirurgiões sendo 4 deles seniores, com mais de 6 anos de experiência, e um júnior com 2 anos de experiência no início do estudo.

**Tabela 1:** Casos

Idade	Sexo	Porta	Indicação	Vancouver	Local	Haste cimentada	Modelo usado	Diagnóstico	Tratamento
74	F	C	Fratura	A2	GT	Não	Stryker-Accolade II	Sim	Conversão + cerclagem
56	M	B	Artrose	A2	GT	Não	Zimmer-ML	Sim	Cerclagem
73	F	A	Artrose	A2	Calcar	Não	Stryker-Accolade II	Sim	Cerclagem
74	F	B	Fratura	A2	Calcar	Não	Jhonson-Corall	Sim	Cerclagem
65	F	B	Artrose	A2	Calcar	Não	Jhonson-Corall	Sim	Conversão + cerclagem
78	F	A	Artrose	A2	GT	Não	Aesculap- Bicontact	Não	RATQ
67	F	B	Artrose	A2	Calcar	Não	Jhonson-Corall	Sim	Conversão + cerclagem
47	M	A	Artrose	A2	Calcar	Não	Aesculap – Bicontact	Sim	Cerclagem
83	M	A	Artrose	A2	Calcar	Não	Jhonson-Corall	Sim	Conversão + cerclagem
62	M	B	Artrose	B2	Diafise	Sim	Baumer-Alpha	Sim	Cerclagem
32	F	A	Artrose	A2	Calcar	Não	Aesculap- Bicontact	Sim	Cerclagem
40	F	B	Artrose	A2	Calcar	Não	Aesculap- Bicontat	Sim	Cerclagem

Abreviaturas: GT, grande trocanter; Conversão: substituição por haste femoral cimentada, RATQ, revisão de artroplastia.

A amostra foi de 2.217 artroplastias realizadas em 2.154 pacientes, tendo sido identificadas 12 fraturas (0,56%). Todas as radiografias do período pós-operatório imediato, como os exames de imagem de acompanhamento subsequentes (15° dia, 4 e 6 semanas, 3 meses, 6 meses e anuais) foram analisadas.

Os dados obtidos foram analisados quantitativamente através do software Microsoft Excel Office 2010 (Microsoft Corporation, Redmond, WA, EUA) para medidas de frequência absoluta e relativa. As comparações entre as variáveis foram realizadas por meio do teste t de Student após verificação da normalidade e da variância dos dados. O significado estatístico foi de *p* < 0,05.

## Resultados

Foram detectadas 12 fraturas do fêmur ocorridas intraoperatoriamente (0,56%) em um total de 2.117 artroplastias do quadril em 2.054 pacientes durante 6 anos ( Tabela 2 ).

**Tabela 2:** Artroplastias realizadas no período de 6 anos pelo grupo de cirurgiões, número total e porcentagem de fraturas periprotéticas intraoperatórias

	Número total de cirurgias	Número total de fraturas periprotéticas	Porcentagem das fraturas periprotéticas
2014	272	0	0%
2015	280	1	0,35%
2016	335	1	0,29%
2017	382	0	0%
2018	440	6	1,36%
2019	408	4	0,98%

O componente femoral foi cimentado em 1.208 casos (57,06%) e não cimentado em 909 casos (42,93%), havendo um aumento da frequência do uso dos implantes não cimentados no decorrer dos anos ( Tabela 3 ).

**Tabela 3:** Quantidade total de componentes femorais cimentados e não cimentados

Ano	Cimentado	Não cimentado
2014	156	116
2015	161	119
2016	176	159
2017	117	265
2018	179	261
2019	120	288

Onze fraturas foram detectadas no ato intraoperatório. Um caso em que não houve diagnóstico durante o ato cirúrgico, nem foi possível evidenciar a fratura na radiografia de controle imediata, o paciente evoluiu com desconforto pós-operatório e dor desproporcionais e no 13° dia apresentou-se ao pronto atendimento relatando dor de forte intensidade associada a impotência funcional sem histórico de trauma, sendo identificada a fratura, que ocorreu no período intraoperatório.

Em relação à cimentação do componente femoral ocorreu 1 caso (0,11%) envolvendo o uso de implante cimentado (n = 909, *p* = 0,004), e 11 casos (0,91%) durante o uso de implantes não cimentados (n = 1.208, *p* = 0,005). Dos 12 casos relatados, 8 ocorreram em mulheres (66,6%), e a idade dos pacientes variou entre 32 e 83 anos, com média de 62,5 anos.

Dez artroplastias ocorreram de maneira eletiva decorrentes de um processo de artrose, apenas duas foram indicadas por fraturas do colo femoral. Embora realizados por cirurgiões diferentes, todos utilizaram a mesma via de acesso póstero-lateral, para todas as hastes a técnica cirúrgica consistiu em fresagens sequenciais do canal até atingir a estabilidade do implante. Nenhuma fratura ocorreu durante as reduções teste. Em 4 casos foi optado por substituição da haste femoral por uma haste cimentada e estabilização da fratura com cerclagem utilizando fio de aço 1,5 mm acima do pequeno trocanter ou envolvendo o grande trocanter quando necessário. Nos demais casos, a haste foi mantida e o tratamento ocorreu utilizando cerclagem com fio de aço 1,5 mm, conforme exemplo na Fig. 1 .

**Fig. 1:** ( **A-B** ) Radiografia pré-operatória, ( **C** ) Radiografia de controle imediato, manutenção da haste e cerclagem acima do pequeno trocanter. Fonte: acervo do pesquisador.

No caso em que a fratura não foi identificada no ato intraoperatório mas no retorno pós-operatório do paciente, na radiografia foi detectada uma fratura com início no grande trocanter e extensão para região metafisária (Vancouver B2) além de sinais compatíveis com a soltura do implante. Neste caso, foi realizada uma artroplastia de revisão com haste femoral de fixação distal ( Fig. 2 ).

**Fig. 2:** ( **A** ) Radiografia de pós-operatório imediato, ( **B** ) Radiografia no 13° dia de evolução, ( **C** ) radiografia de controle após revisão. Fonte: acervo do pesquisador.

Foi obtida estabilidade visual e tátil em todos os casos, sendo permitida descarga de peso pelo paciente conforme o tolerado.

## Discussão

A fratura periprotética intraoperatória do fêmur é uma complicação muito descrita na literatura e carece de estudos com ampla amostragem. Quando não detectada no ato, acarreta em falha precoce do implante, aumento do tempo de internamento, aumento da morbidade e dos custos inerentes ao procedimento. 10 Esta possui uma incidência variável na literatura, sendo de aproximadamente 0,62% para implantes cimentados e 0,87% para implantes não cimentados, em uma amostragem de 793.823 ATQs. 11 Atualmente, ocorre um aumento da incidência deste evento que está relacionado ao envelhecimento da população e ao aumento do uso de hastes não cimentadas. 12 13

No decorrer de 6 anos, obtivemos uma incidência de 0,11% utilizando hastes cimentadas e 0,91% utilizando hastes não cimentadas, dados compatíveis com a literatura mundial. O risco aproximadamente dobra em mulheres; 14 nossos dados apontam para um aumento da prevalência de fraturas com um aumento da idade nas mulheres, o que não ocorre com os homens. Tal diferença pode existir porque as mulheres são mais afetadas pela osteoporose pós-menopausa, o que gera diminuição da resistência óssea. 15

O aumento da idade já foi associado ao aumento de fraturas intraoperatórias; 14 porém, os pacientes jovens podem ter um risco maior a sofrerem fraturas do calcar devido ao canal femoral proximal ser mais estreito e necessitar de uma fresagem mais prolongada e com maior força transmitida entre o osso e o instrumental.

A análise da Tabela 2 nos mostra o aumento progressivo da utilização de hastes femorais não cimentadas em detrimento das cimentadas, uma mudança que aconteceu no decorrer dos anos em função de uma tendência global na mudança da técnica. Hastes não cimentadas, quando devidamente indicadas e executadas, podem diminuir o tempo cirúrgico e também as chances de embolia pulmonar decorrentes do processo de cimentação. 16 Outra possível explicação desta migração deve-se ao fato das menores taxas de revisão devido à soltura asséptica. 17

O modelo de haste não cimentada é o mais comum nas ATQs realizadas nos Estados Unidos, embora os estudos demonstrem excelentes resultado ao longo dos anos utilizando diferentes opções de fixação femoral, cenário que aponta para um aumento do risco de fratura intraoperatória no uso das hastes não cimentadas, 14 como evidenciado em nossos dados. O correto planejamento da prótese com radiografias e com transparências é fundamental para diminuir esse risco, no qual existe a possibilidade de que parâmetros radiográficos específicos indiquem um risco maior de fratura periprotética, como por exemplo, os que possuem conformação Dorr tipo A e tipo B. Dentro de nossa amostragem, 42% das fraturas ocorreram em canais tipo B e 50% em canais tipo A.

As fraturas do calcar ou da diáfise tendem a ocorrer durante a preparação do canal ou inserção do implante, aonde a maioria das hastes não cimentadas utiliza o conceito de *pressfit* que aumenta a tensão na cortical óssea. Dentre as causas de fraturas relacionadas a condições cirúrgicas inerentes ao ato operatório ocorridas na região proximal podemos citar a desproporção entre a fresa utilizada no preparo e o implante definitivo, também chamado de *mismatch* , ou pelo uso excessivo da força durante a exposição cirúrgica e ou no preparo do canal femoral. Por fim, as fraturas distais geralmente ocorrem por colisão da ponta reta do implante com a cortical curva do fêmur. O tipo de fratura em que o pequeno trocanter é acometido ocorre secundário à propagação de microfraturas que podem ter ocorrido durante a lesão primária, no caso de uma fratura, ou durante a osteotomia. Já o padrão ao redor da haste ocorre devido à incompatibilidade da junção metáfise-diafisária da haste e o canal durante a implantação em fêmures osteoporóticos. 18

A durabilidade da haste não cimentada demonstrou ser maior em pacientes jovens talvez devido à melhor qualidade óssea, o que diminui o risco de fraturas. 19 Porém, o aumento do risco de fratura intraoperatória e, consequentemente, suas complicações devem ser ponderados em relação à maior sobrevida do implante, principalmente nos pacientes nos quais o fêmur proximal parece fraco ou nos casos que exigem preparo prolongado e maior utilização de força, como nos casos Dorr A. 11

A avaliação do design da haste é algo que sempre deve ser levado em consideração, já que a utilização de hastes em cunha revela um risco 10 vezes maior de causar fratura intraoperatória quando comparada às hastes de primeira geração. 20 Em dois casos apresentados neste estudo foram utilizados implantes femorais de cunha simples com o conceito de *Fit and Fill* (Accolade II – Stryker Corporation, Kalamazoo, MI, USA), este design está associado à um aumento de 3 vezes nas taxas de fratura intraoperatória ( *p* < 0,001) em comparação com as hastes anatômicas, totalmente revestidas e cônicas ou arredondadas. 21 Quatro casos apresentados ocorreram utilizando hastes de preservação como a *Corail* ou *Bicontact,* sobre as existe um estudo que relaciona o modelo da haste com um maior risco de fratura. O modelo de raspas, no qual ocorre a impacção óssea metafisária que resulta em preservação óssea, aumentaria a tensão ao redor do canal no momento da introdução do implante definitivo, aumentando assim as chances de fraturas intraoperatórias. 22

É importante analisar se a indicação da artroplastia, por fratura ou artrose, interfere na ocorrência de fraturas intraoperatórias. Em nosso estudo, em apenas 2 casos (16,6%) a indicação foi por fratura do colo e em 10 casos (83,3%) por artrose. A literatura é controversa neste ponto. Os que advogam a favor do uso de hastes cimentadas usam como argumento o aumento do risco de fratura intraoperatória devido à fragilidade óssea pré-existente que teria culminado com a fratura do colo, em paralelo ao fato de que a utilização de hastes cimentadas poderia aumentar a mortalidade, por complicações decorrentes da cimentação femoral, e o aumento do tempo cirúrgico. 16

Klein et al., 23 em 2006, em um estudo que reuniu 85 casos de fraturas de colo, com idade média de 78 anos, tratados com haste não cimentada, demonstraram que não há um aumento significativo no caso de fraturas intraoperatórias e que os pacientes apresentaram boa recuperação funcional. Richardson et al., 24 em 2020, com a análise de 5.883 casos de artroplastia total em fraturas de colo, compararam as taxas de mortalidade nas duas técnicas e obtiveram como resultado a diminuição nas taxas de mortalidade ao utilizar hastes cimentadas com 30, 90 e 365 dias após a cirurgia. Esta diferença se deve principalmente à ocorrência de fraturas intraoperatórias e, consequentemente, reoperações quando utilizadas hastes não cimentadas. Ekman et al., 25 em 2019, estudaram os efeitos da cimentação em 10.677 artroplastias totais e parciais, concluindo que não há diferença na mortalidade. A literatura ainda é muito controversa neste ponto e carece de mais estudos.

Considerando que a maior complicação da fratura intraoperatória é justamente não a diagnosticar no ato cirúrgico, é importante atentar para os sinais desta intercorrência. Ela é definida como a fratura identificada durante o procedimento cirúrgico ou diagnosticada na radiografia de controle imediata; alguns sinais, como a mudança súbita de resistência durante a inserção do componente definitivo, parada do implante definitivo abaixo do nível da osteotomia, ruído audível, implante definitivo com tamanho diferente do planejado e ou perda da estabilidade, nos sugerem uma eventual fratura. 5

No geral, o tratamento das fraturas periprotéticas agudas está relacionada a um alto índice de complicações (63%) e reoperações (23%), e o diagnóstico intraoperatório resulta em uma alta resolução favorável e com baixa complexidade do procedimento. A tática consiste em aplicação de uma cerclagem circunferencial com fio de aço acima do pequeno trocanter e uso do mesmo implante após a cerclagem com estabilização da fratura. 9

Neste estudo, a falha do diagnóstico intraoperatório gerou a necessidade de uma artroplastia de revisão no 13° dia de pós-operatório. A revisão dos exames mostrou tratar-se de uma fratura oculta, que após intervenção evoluiu com a consolidação da fratura e total recuperação funcional. Em três casos, houve a troca do implante não cimentado por uma haste cimentada e associação de cerclagem no trocanter, sendo esta opção devido à maior fragilidade óssea femoral, detectada pelo cirurgião. Todos os casos evoluíram com consolidação óssea, recuperação funcional total do paciente, e sem a soltura do implante no decorrer dos anos.

Quando diagnosticada intraoperatoriamente e tratada com a técnica da cerclagem acima do pequeno trocanter, a fratura periprotética possui alta taxa de resolutividade e garantia de um bom desfecho. Já o diagnóstico tardio acarreta um tratamento mais complexo e com maiores chances de complicações, quando devemos utilizar a classificação de Vancouver para fraturas periprotéticas pós-operatórias, que norteará a estratégia de tratamento mais adequada. 26

Assim, o desenvolver de habilidades pelo cirurgião, como reconhecer uma morfologia femoral específica, pode alterar o planejamento cirúrgico e mesmo a seleção da haste femoral. 27

Este estudo fornece informações sobre a incidência, riscos e fatores associados à fratura periprotética intraoperatória do fêmur durante a ATQ. Entender como ela ocorre e identificar possíveis fatores que indiquem tal complicação é fundamental para que possamos diminuir as complicações, pois uma vez diagnosticada intraoperatoriamente esta possui um tratamento menos complexo e com melhores resultados.

## Conclusões

A análise dos dados permite afirmar que o risco de fratura periprotética intraoperatória é maior em mulheres e com o uso de hastes não cimentadas. A ocorrência destas neste estudo teve taxa inferior a 1%.
